# Supplementary material for: Implementing Systems Modelling and Molecular Imaging to Predict the Efficacy of BCL-2 Inhibition in Colorectal Cancer Patient-Derived Xenograft Models
Source: Cancers (Basel). 2020 Oct 14;12(10):2978. doi: 10.3390/cancers12102978 (PMC7602510; doi:10.3390/cancers12102978)
Supplement: Supplementary file 1 [file cancers-12-02978-s001.pdf]

Supplementary Materials

Implementing Systems Modelling and Molecular Imaging to Predict the Efficacy of BCL-2 Inhibition in Colorectal Cancer Patient-Derived Xenograft Models

Alice C. O'Farrell, Monika A. Jarzabek, Andreas U. Lindner, Steven Carberry, Emer Conroy, Ian S. Miller, Kate Connor, Liam Shiels, Eugenia R. Zanella, Federico Lucantoni, Adam Lafferty, Kieron White, Mariangela Meyer Villamandos, Patrick Dicker, William M. Gallagher, Simon A. Keek, Sebastian Sanduleanu, Philippe Lambin, Henry C. Woodruff, Andrea Bertotti, Livio Trusolino, Annette T. Byrne and Jochen H.M. Prehn

Table S1. BCL-2 protein profiles by quantitative Western Blotting.

ID	*Type*	*Method*	*Source*	*BAK(nM)*	*BAX (nM)*	*BCL2 (nM)*	*BCLxL (nM)*	*MCL1 (nM)*	*DR_MOMP (nM)*	*Comment*
DLD-1	cell line	Western Blot	new	779.2	409.7	22.2	199.3	11.6	512.7	p53 mutant
HCT-116	cell line	Western Blot	Cancer Research, 2013	676.7	317.6	230.6	604.4	25.3	666.3	p53 wildtype
CRC0344	PDX	Western Blot	new	1,009.2	1,475.1	1,979.7	1,248.5	301.9	2,601.4	
CRC0076	PDX	Western Blot	new	1,190.9	2,155.5	5,771.9	567.6	336.9	4,270.6	

Table S2. Stress dose' calculated using DR_MOMP.

	DR_MOMP [nM] for # nM ABT-199 (Venetoclax)					
ID	***0***	***100***	***500***	***1,000***	***5,000***	***10,000***
DLD-1	512.7	476.8	386.4	312.6	145.9	105.8
HCT-116	666.3	647.3	565.8	444.7	93.3	53.1
CRC0344	2,601.4	2,574.3	2465.4	2,328.6	1156.1	449.7
CRC0076	4,270.6	4,244.2	4138.7	4,005.8	2911.6	1,506.1

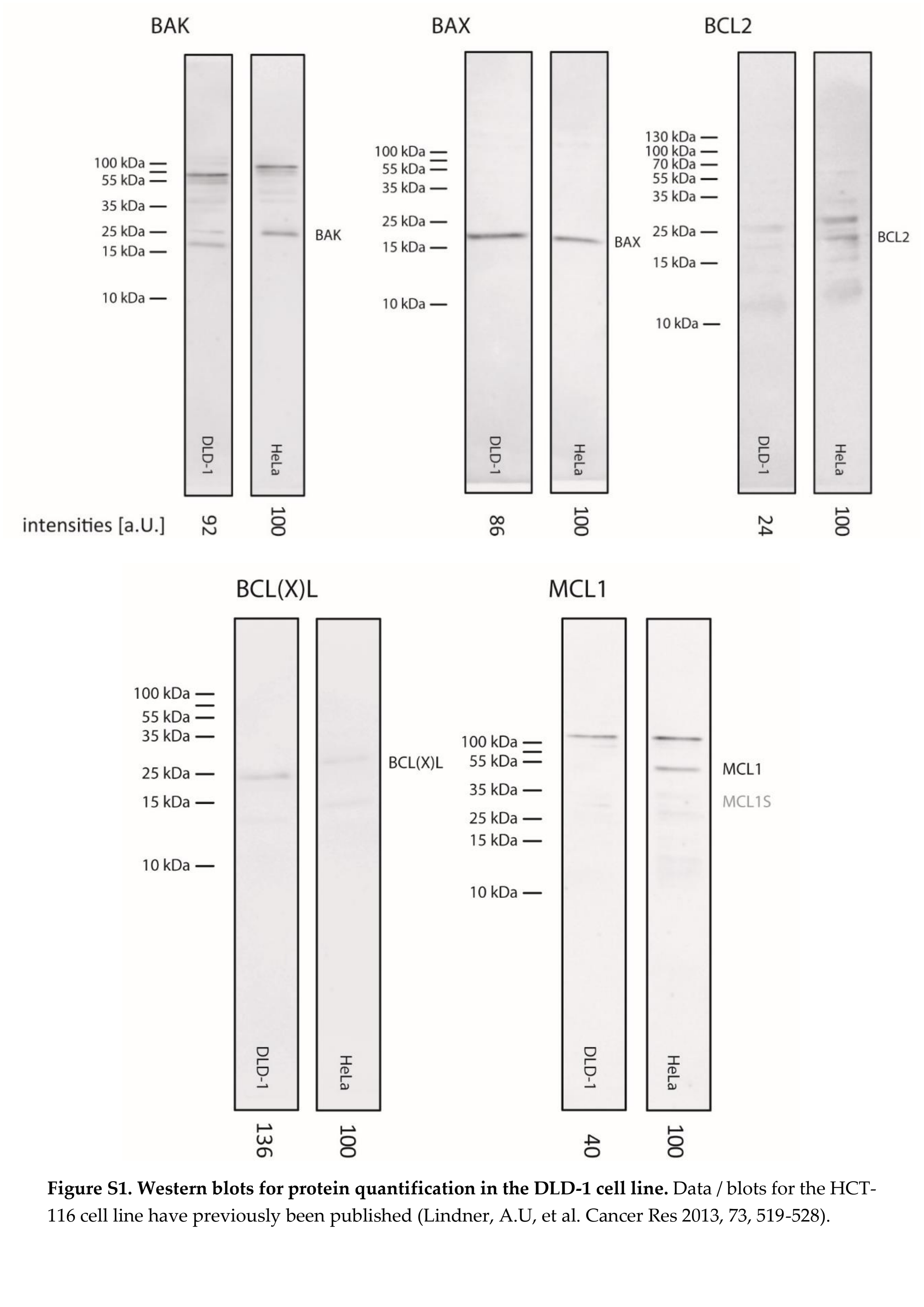

Figure S1. Western blots for protein quantification in the DLD-1 cell line. Data / blots for the HCT-116 cell line have previously been published (Lindner, A.U, et al. Cancer Res 2013, 73, 519-528).

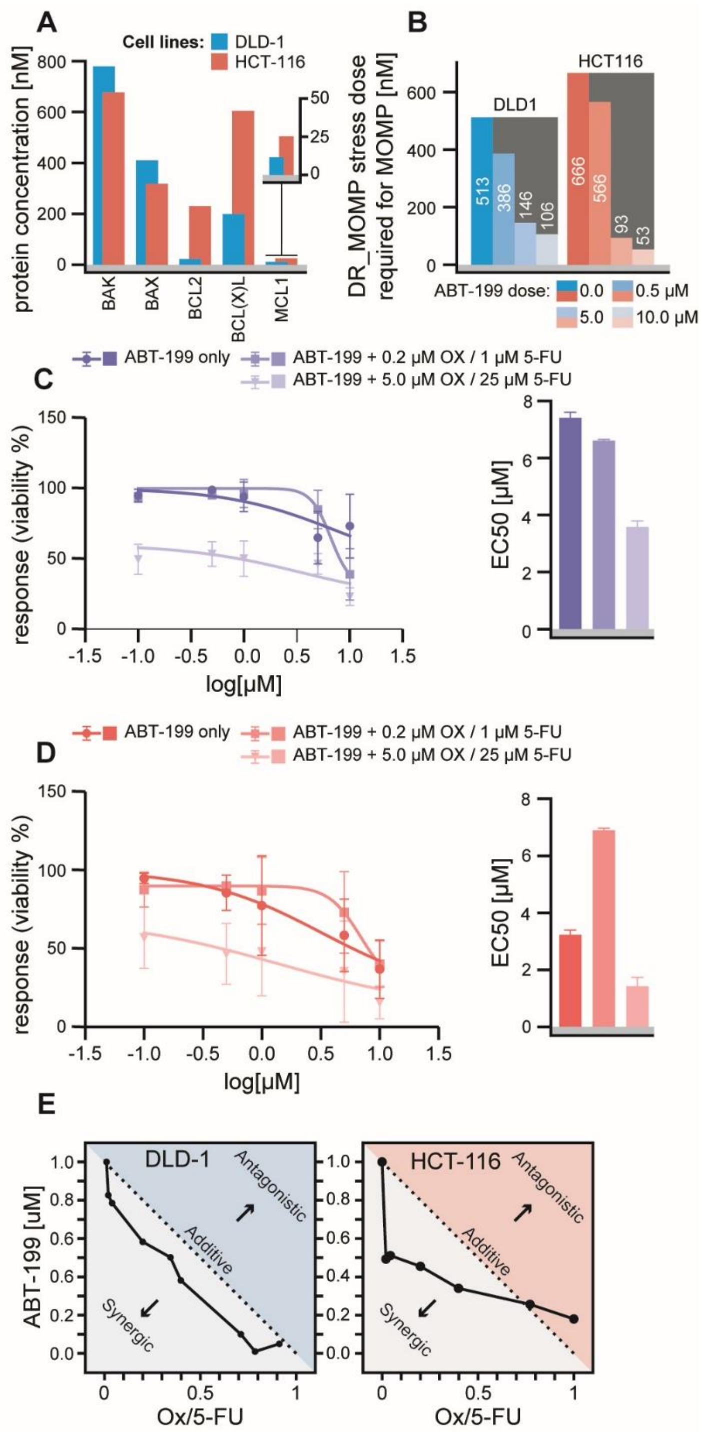

Figure S2. A synergistic effect of ABT-199 and 5-FU/OX combination observed in CRC cell lines. BCL-2 family protein levels were quantified in the p53 mutated DLD-1 (blue) and p53 wild-type HCT-116 (red) cell lines using Western blot (A) and their susceptibility to combination treatment with ABT-199 and genotoxic stress dose was calculated using DR_MOMP (B) based on protein profiles from Lindner et al. 2013 (7). (C, D) A 6 × 6 dose matrix assay was performed by treating DLD-1 (C) and HCT-116 cells (D) with increasing concentrations of 5-FU/OX (with a concentration ratio of 1:5 between the OX and 5-FU drugs) in combination with increasing concentrations of ABT-199. Cell survival was assessed after 48 h treatment with an acid phosphatase assay. Experiments were performed in triplicates (2 wells each repetition). Represented are dose-response curves and EC50 values calculated with Prism (GraphPad, La Jolla, CA) by using a nonlinear regression with a variable slope fit function. E) Isobologram analysis for fraction affected % calculated from the 6 × 6 dose matrix assay results for DLD-1 cells (blue) and HCT-116 (red), respectively.

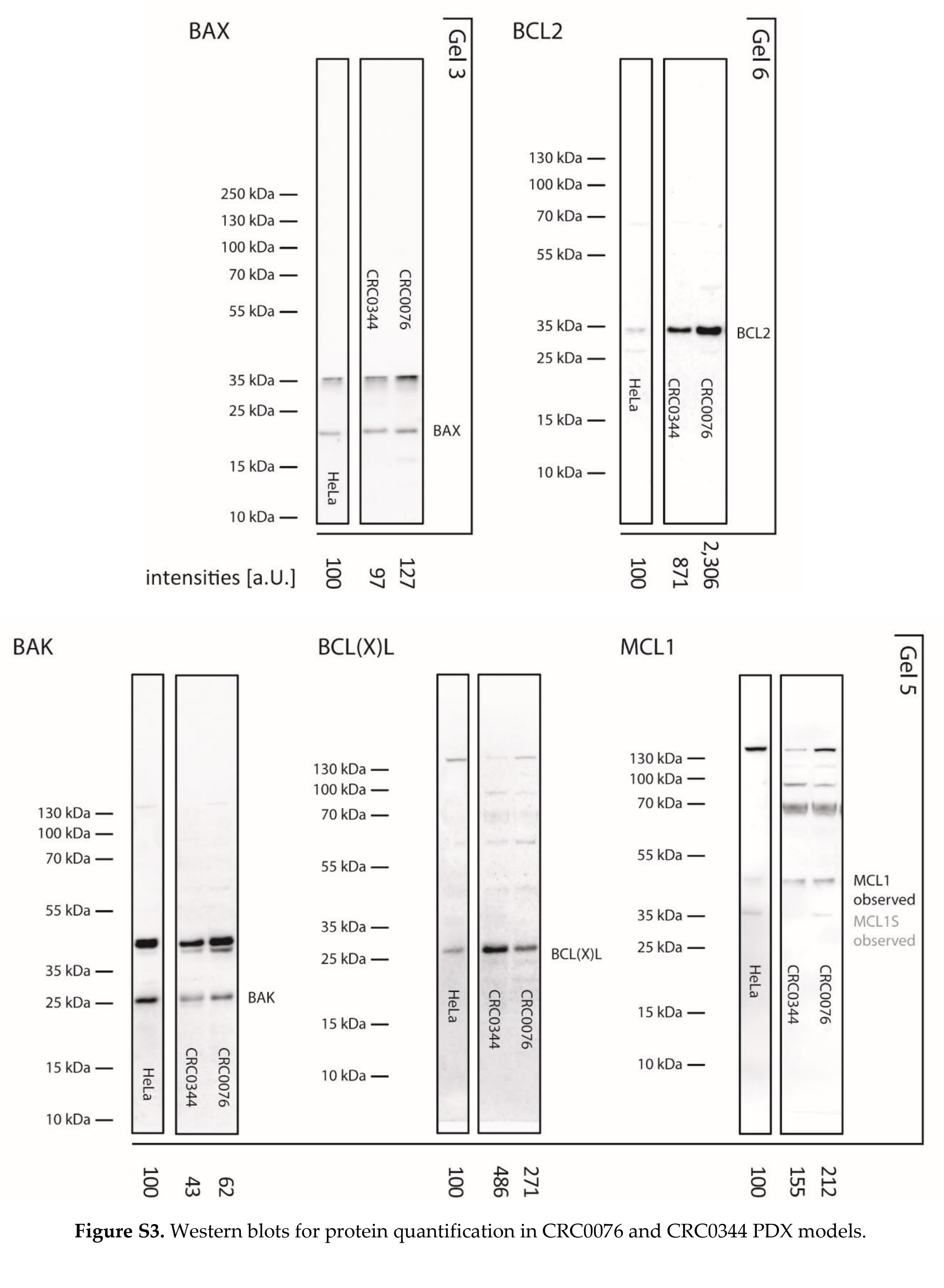

Figure S3. Western blots for protein quantification in CRC0076 and CRC0344 PDX models.

A) PUMA – CRC0344

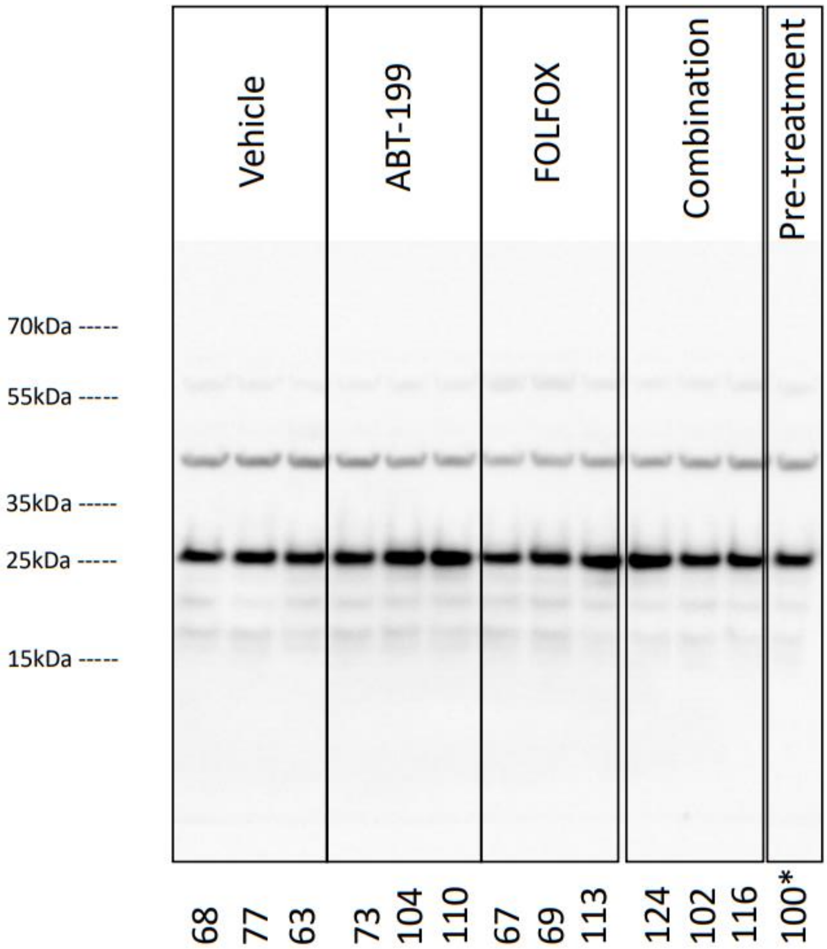

*Intensities given are after normalisation: All band intensities are normalised to pre-treatment, following application of a correction factor for loading – please refer to next slide for loading controls.

B) ACTIN – CRC0344 (for PUMA)

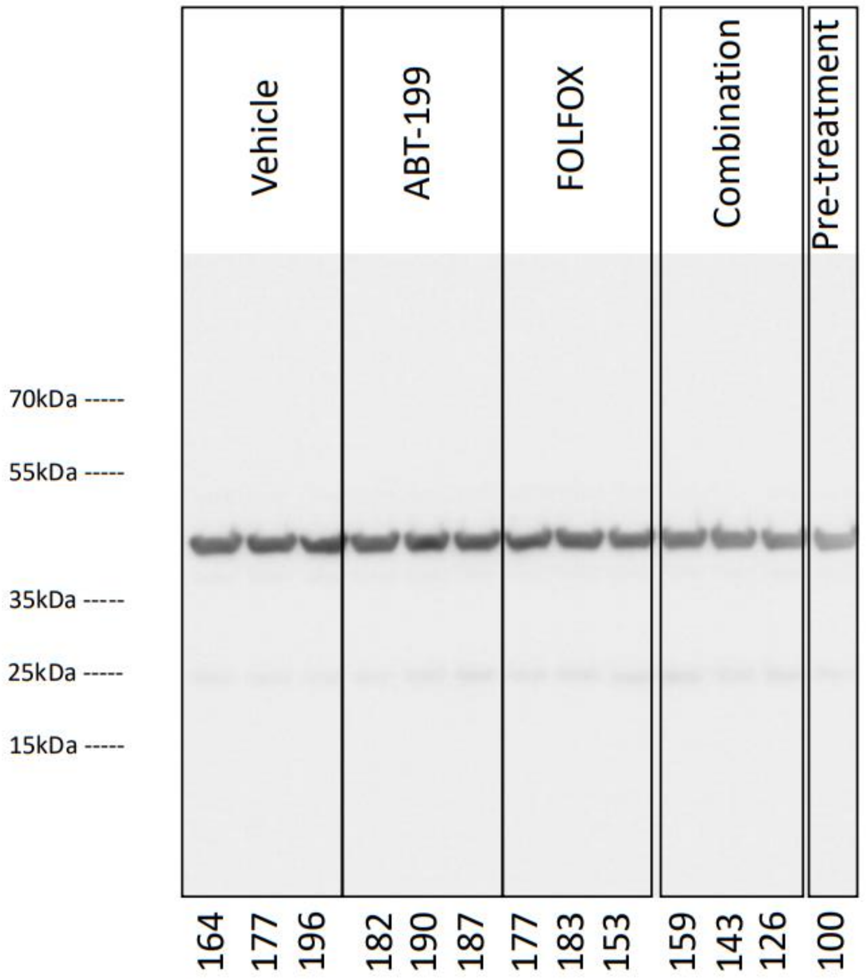

C) BIM – CRC0344

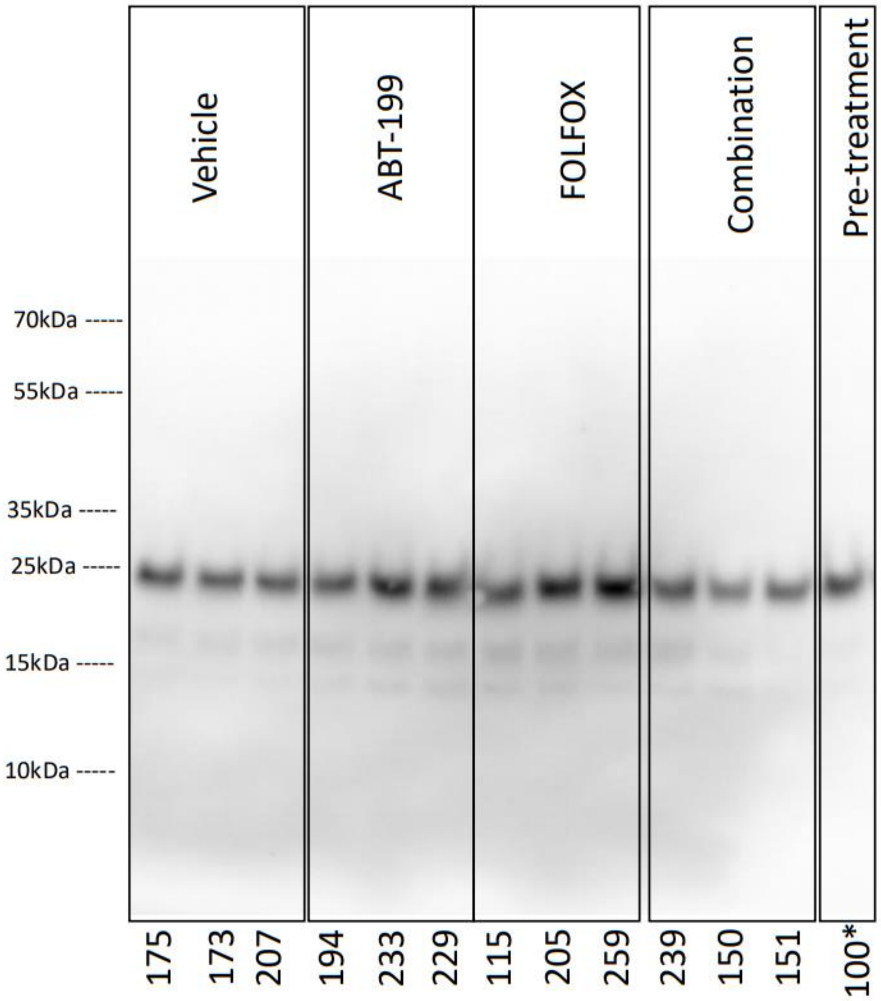

D) GAPDH – CRC0344 (for BIM)

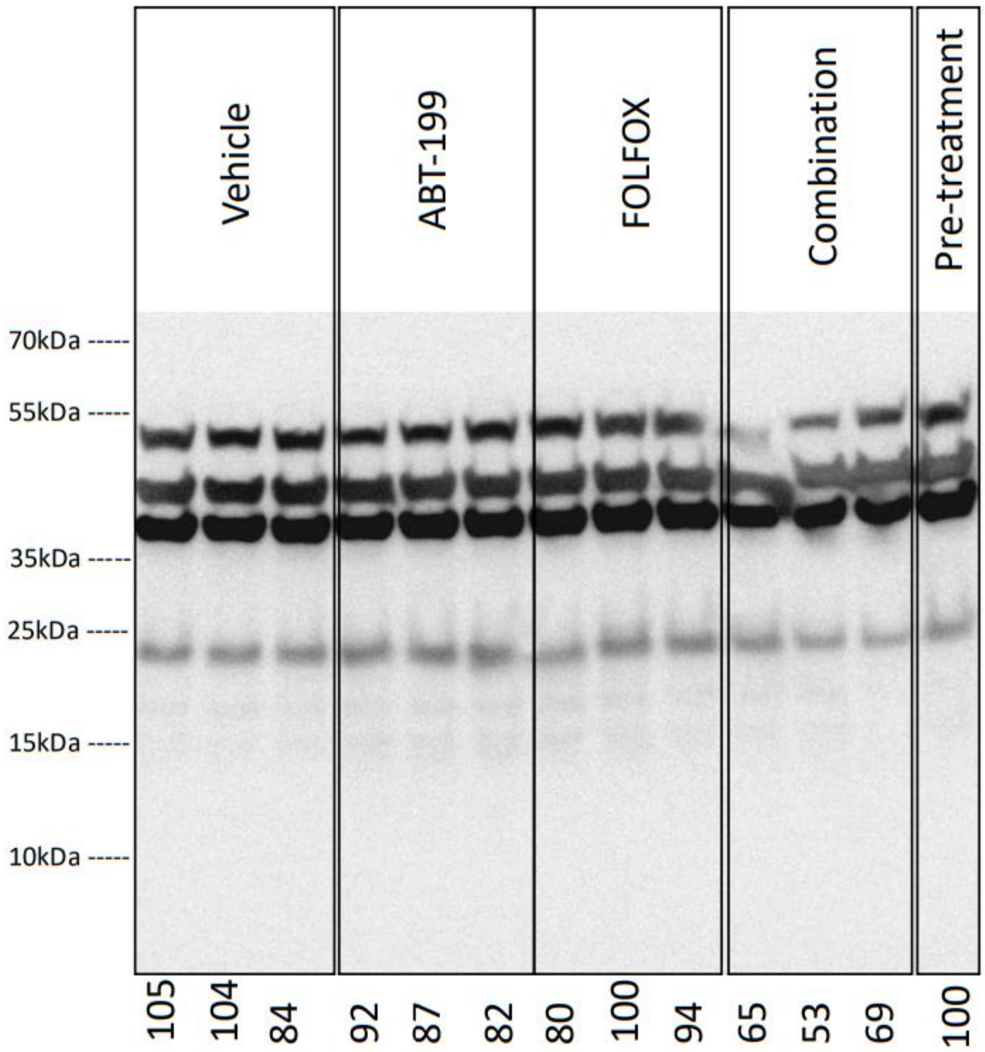

E) PUMA – CRC0076

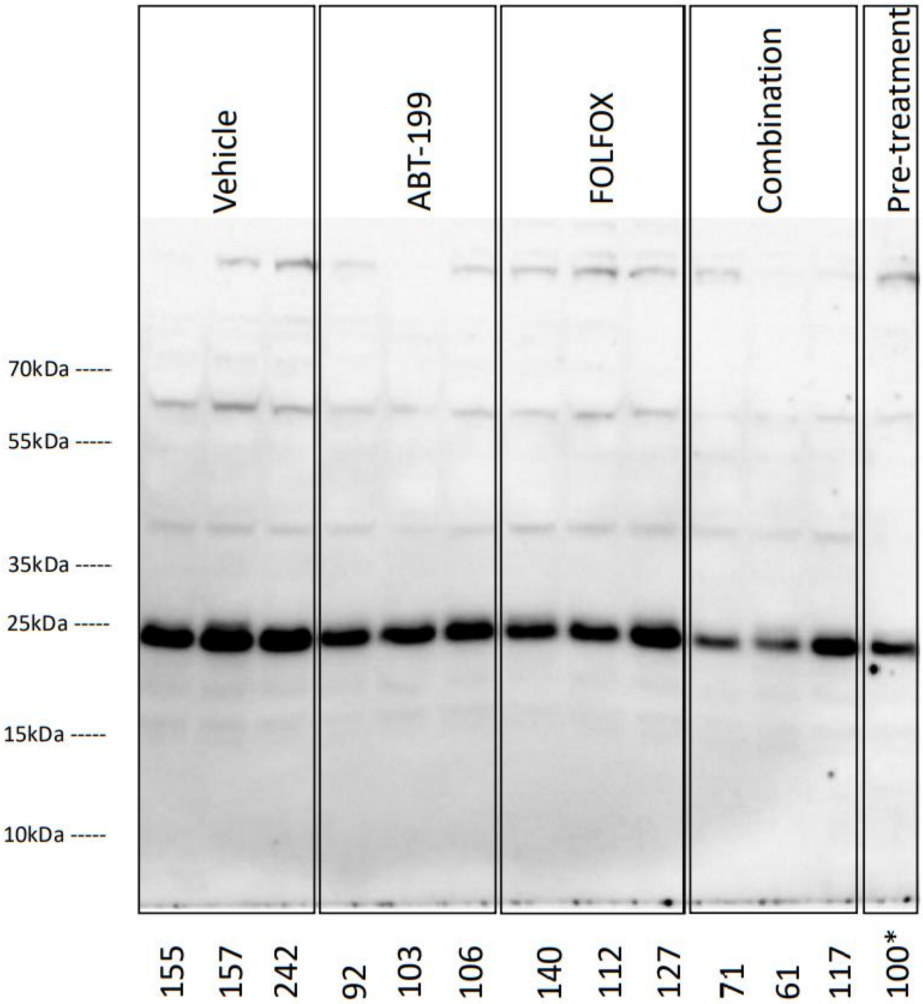

*Intensities given are after normalisation: All band intensities are normalised to pre-treatment, following application of a correction factor for loading – please refer to next slide for loading controls.

F) ACTIN – CRC0076 (for PUMA)

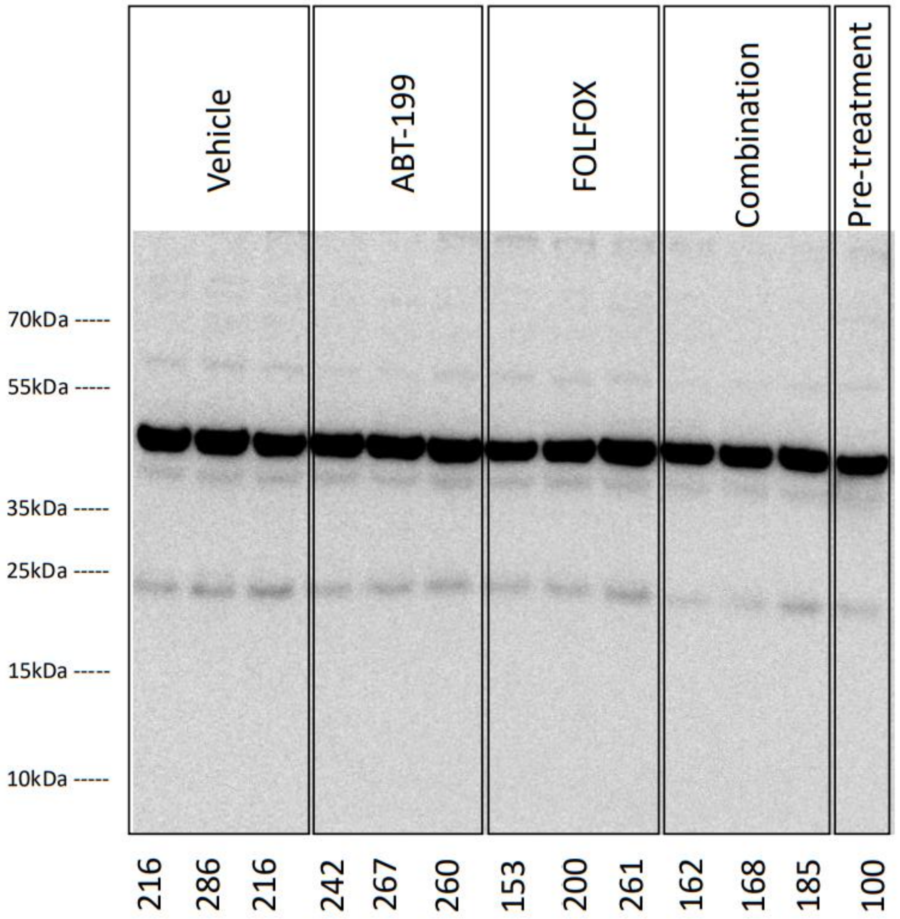

G) BIM – CRC0076

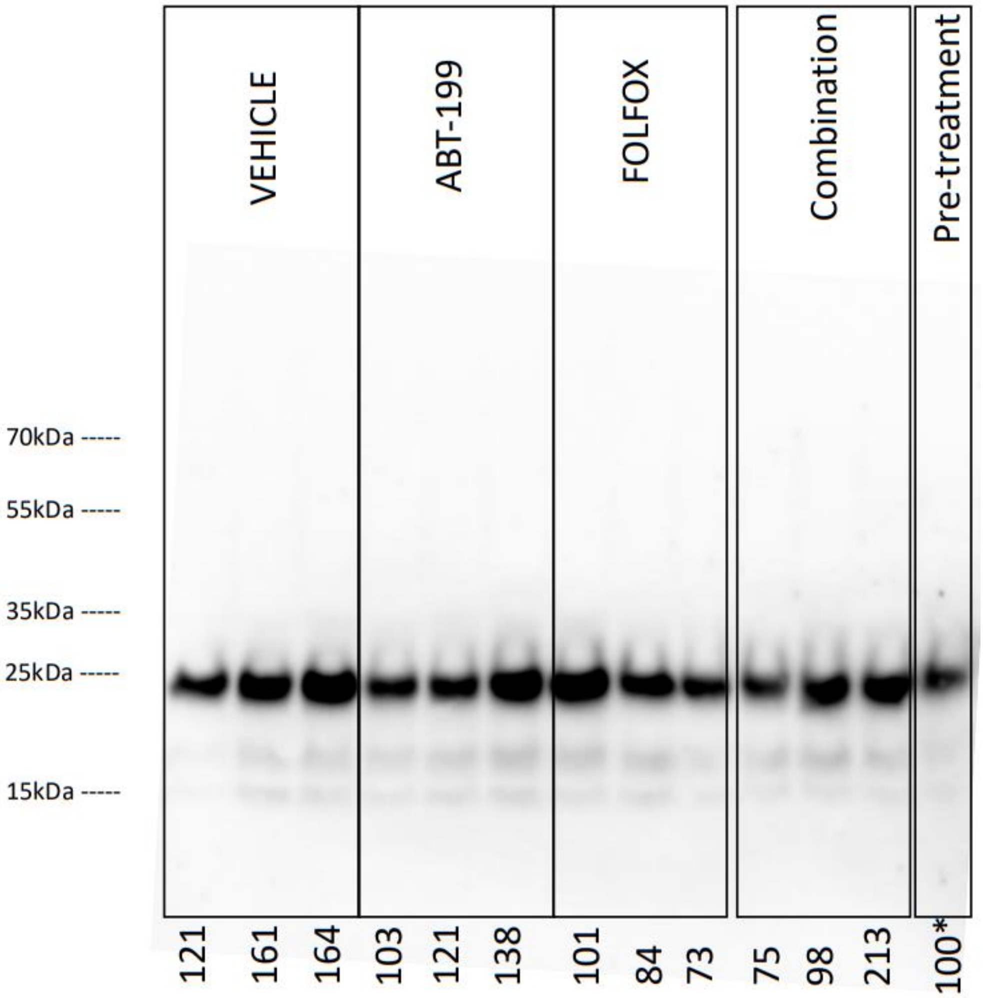

*Intensities given are after normalisation: All band intensities are normalised to pre-treatment, following application of a correction factor for loading – please refer to next slide for loading controls.

H) ACTIN – CRC0076 (for BIM)

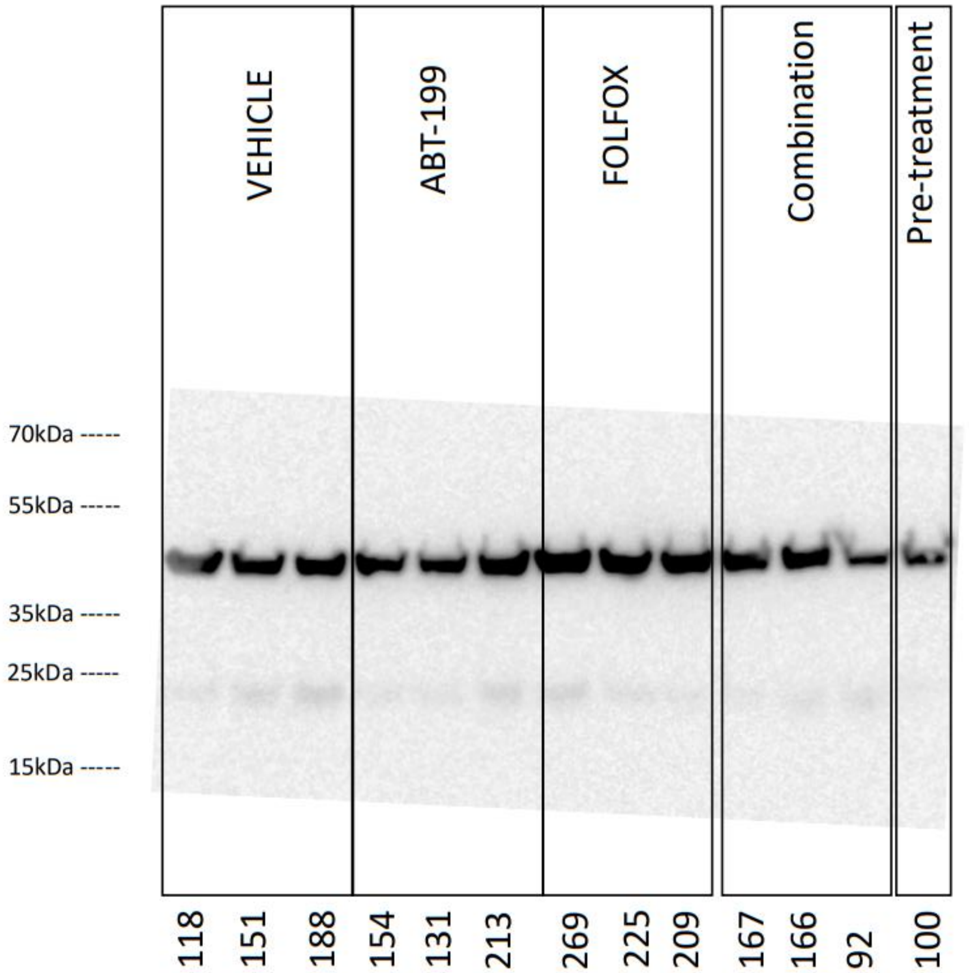

Figure S4. Full BIM and PUMA Western blots undertaken in post-mortem CRC0076 and CRC0344 tumours.

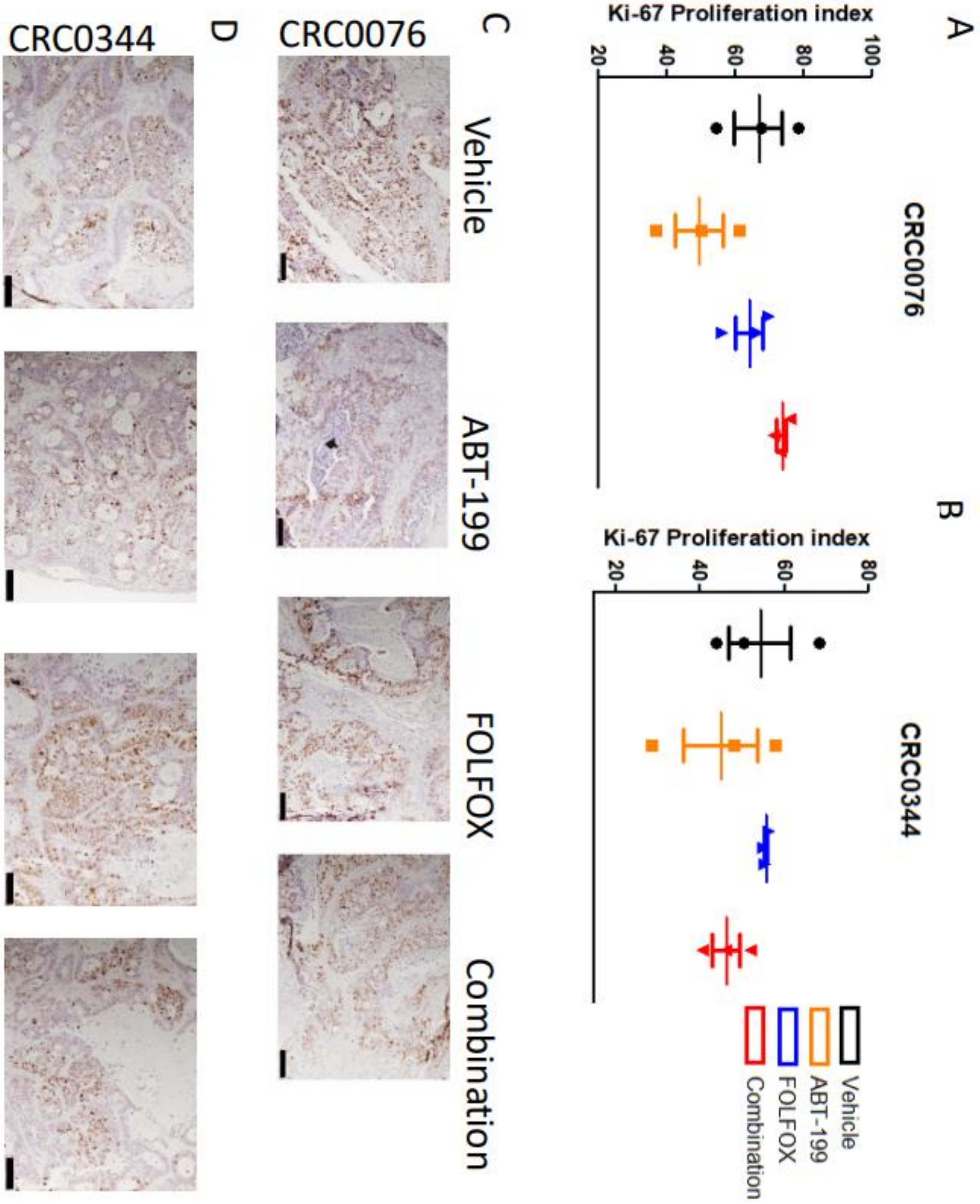

Figure S5. Ki-67 analysis of CRC0076 and CRC0344 PDX tumours after 28 days of treatment**.** Analysis of Ki-67 proliferation index in CRC0076 (A) and CRC0344 (B) PDX models. Representative image from all treatment groups in CRC0076 (C) and CRC0344 (D); Magnification 10x, scale bar 100 μm. Error bars represent mean ± s.e.m. (*n* = 3 tumours per treatment, 8-10 images per section analysed). No significant differences apparent between any treatment groups (One-way ANOVA, adjusted p-values ≥ 0.05 [Tukey's multiple comparisons test]).

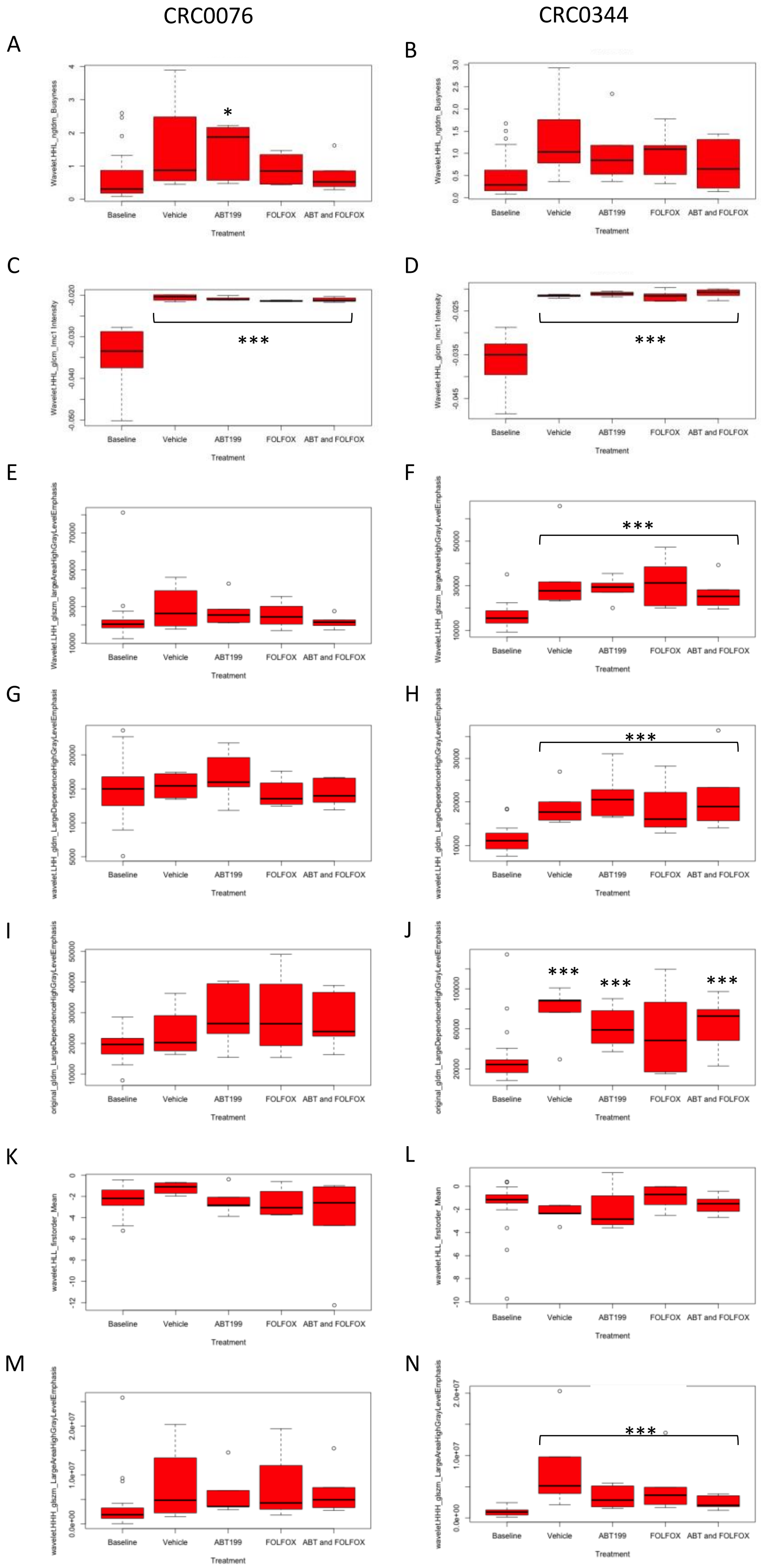

Figure S6. Evaluation of radiomics features as early biomarkers of response to FOLFOX and ABT-199 combination therapy in PDXs CRC0076 and CRC0344. The 7 radiomics features elucidated from pre-treatment CT images of PDX CRC0076 (A, C, E, G, I, K, M) and CRC0344 (B, D, F, H, J, L, N) were extracted from post-treatment CT scans. Changes in the intensity of individual features after 2 weeks of treatment were compared to pre-treatment * $p < 0.05$ ***$p < 0.001$ Wilcoxon Rank sum test error bars

represent SEM CRC0076: Vehicle *n* = 4, ABT199 *n* = 5, FOLFOX *n* = 4, ABT+FOLFOX *n* = 6 CRC0344: Vehicle *n* = 5, ABT199 *n* = 6, FOLFOX *n* = 6, ABT+FOLFOX *n* = 5).

Document S1.

R code used to develop the radiomic classifier to separate PDX CRC0076 and PDX CRC0344
R version 3.6.1

Dependencies for Script: gplot, ggplot2, rms, pROC, PredictABEL, sm, stats, base, caret, ROSE, xgboost, tidyr, dplyr, e1071, skimr, DataExplorer

```
BaselineNS<-read.csv('insert file name")

BaselineNS <-  BaselineNS[, colMeans(is.na(BLforDR)) == 0]
badCols <- nearZeroVar(BLforDR)
if (length(badCols > 0)){
  Baselineall <- Baselineall[, -badCols]
}
length(badCols)

#Partitioning
PartitionNS <-createDataPartition(y=BaselineNS$PDX,p=0.8, list=FALSE)

#Training
TrainNS<-BaselineNS[PartitionNS,]
OutcomeTrainNS<-TrainNS$PDX

#Validation
ValNS<-BaselineNS[-PartitionNS,]
OutcomeValNS<-ValNS$PDX

#correlation
corMAtrix=cor(TrainNS[,1:806], method=c('spearman'))
highly_correlated_columns = findCorrelation(corMAtrix, cutoff=0.85)
Train_uncor_NS <- TrainNS[,-highly_correlated_columns]

#Recursive feature elimination

Functions<- treebagFuncs
Repeats<- 3

Methods<- "repeatedCV"
Sizes<- c(1:10)
ctrl <- rfeControl(functions = Functions, method = Methods, repeats = Repeats, number = 10, verbose
= T)
RFEProfile <- rfe(x= Train_uncor_NS, y= as.factor(OutcomeTrainNS), sizes= Sizes, rfeControl = ctrl)

plot(RFEProfile,type=c("g","o"))
```

```
selectedData_NS<-Train_uncor_NS[,RFEProfile$optVariables[c(1:7)]]
SelectedVal_NS<-ValNS[,RFEProfile$optVariables[c(1:7)]]

#Train the model

N<- 100 #number of boosting iterations.
ml <- xgboost(data=data.matrix(selectedData_NS), label=OutcomeTrainNS, missing = NULL,
params = list (), nrounds=N)

#AUC
predctions_Xg_training <- predict(ml, data.matrix(selectedData_NS))
RocTrain_Xg <-roc(OutcomeTrainNS, predctions_Xg_training)
predctions_Xg_test <- predict(ml, newdata=data.matrix(SelectedVal_NS))
RocTest_Xg <-roc(OutcomeValNS, predctions_Xg_test, ci=TRUE)
```

© 2020 by the authors. Licensee MDPI, Basel, Switzerland. This article is an open access article distributed under the terms and conditions of the Creative Commons Attribution (CC BY) license (http://creativecommons.org/licenses/by/4.0/).
